# Poly[[bis­{μ_2_-1,2-bis­[(1*H*-imidazol-1-yl)meth­yl]benzene}(μ_4_-9,10-dioxo-9,10-dihydro­anthracene-1,4,5,8-tetra­carbox­yl­ato)dicobalt(II)] dihydrate]

**DOI:** 10.1107/S160053681202716X

**Published:** 2013-02-06

**Authors:** Xiao-Li Sheng, Duo-Hui Xu, Bin Cai, Jian-Lan Liu

**Affiliations:** a Department of Applied Chemistry, College of Science, Nanjing University of Technology, Nanjing 210009, People’s Republic of China

## Abstract

The title complex, {[Co_2_(C_18_H_4_O_10_)(C_14_H_14_N_4_)_2_]·2H_2_O}_*n*_ was synthesized from CoCl_2_·6H_2_O, 9,10-dioxo-9,10-dihydro­anthracene-1,4,5,8-tetra­carb­oxy­lic acid (H_4_AQTC) and 1,2-bis­[(1*H*-imidazol-1-yl)meth­yl]benzene (*o*-bix) in water. The anthraquinone unit is located about a crystallographic center of inversion. Each asymmetric unit therefore contains one Co^II^ atom and one *o*-bix ligand, as well as half an AQTC^4−^ ligand and an additional solvent water mol­ecule. The Co^II^ ions are tetra­hedrally surrounded by two O atoms from two AQTC^4−^ anions and by two N atoms from two *o*-bix ligands, forming a two-dimensional coordination polymer. The solvent water mol­ecules are connected to the carboxyl­ate groups by O—H⋯O hydrogen bonds. Additional weak C—H⋯O hydrogen bonds are observed in the crystal structure.

## Related literature

For general background to metal organic frameworks, see: Li *et al.* (1999 ▶, 2012 ▶); Cheng *et al.* (2010 ▶); Hong *et al.* (2009 ▶); Miller & Gatteschi (2011 ▶); Liu *et al.* (2010 ▶).
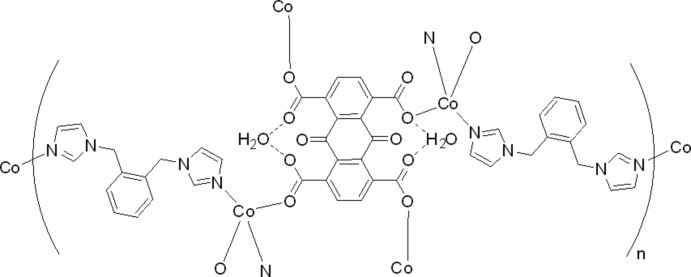


## Experimental

### 

#### Crystal data

[Co_2_(C_18_H_4_O_10_)(C_14_H_14_N_4_)_2_]·2H_2_O
*M* *_r_* = 1010.68
Triclinic, 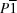

*a* = 9.561 (4) Å
*b* = 10.594 (5) Å
*c* = 12.436 (5) Å
α = 107.095 (7)°
β = 102.454 (6)°
γ = 106.551 (6)°
*V* = 1090.7 (8) Å^3^
*Z* = 1
Mo *K*α radiation
μ = 0.83 mm^−1^
*T* = 296 K
0.43 × 0.36 × 0.28 mm

#### Data collection

Bruker SMART CCD area-detector diffractometer
Absorption correction: multi-scan (*SADABS*; Bruker, 2000 ▶) *T*_min_ = 0.717, *T*_max_ = 0.801
10020 measured reflections
3787 independent reflections
3411 reflections with *I* > 2σ(*I*)
*R*_int_ = 0.045

#### Refinement

*R*[*F* ^2^ > 2σ(*F* ^2^)] = 0.032
*wR*(*F* ^2^) = 0.091
*S* = 1.04
3787 reflections
315 parameters
H atoms treated by a mixture of independent and constrained refinement
Δρ_max_ = 0.39 e Å^−3^
Δρ_min_ = −0.32 e Å^−3^

### 

Data collection: *SMART* (Bruker, 2000 ▶); cell refinement: *SAINT* (Bruker, 2000 ▶); data reduction: *SAINT*; program(s) used to solve structure: *SHELXS97* (Sheldrick, 2008 ▶); program(s) used to refine structure: *SHELXL97* (Sheldrick, 2008 ▶); molecular graphics: *SHELXTL* (Sheldrick, 2008 ▶); software used to prepare material for publication: *SHELXTL*.

## Supplementary Material

Crystal structure: contains datablock(s) I, global. DOI: 10.1107/S160053681202716X/im2380sup1.cif

Structure factors: contains datablock(s) I. DOI: 10.1107/S160053681202716X/im2380Isup2.hkl

Additional supplementary materials:  crystallographic information; 3D view; checkCIF report

## Figures and Tables

**Table 1:** Hydrogen-bond geometry (Å, °)

*D*—H⋯*A*	*D*—H	H⋯*A*	*D*⋯*A*	*D*—H⋯*A*
O6—H24⋯O4^i^	0.83 (5)	2.10 (5)	2.911 (4)	166 (5)
O6—H25⋯O2^ii^	0.99 (6)	1.92 (6)	2.883 (4)	164 (5)
C11—H11⋯O6^iii^	0.93	2.37	3.192 (4)	148
C12—H12⋯O5^ii^	0.93	2.39	3.268 (4)	157
C13—H13*A*⋯O1^ii^	0.97	2.58	3.389 (4)	141
C13—H13*B*⋯O3^iv^	0.97	2.54	3.278 (3)	133
C20—H20*A*⋯O2^ii^	0.97	2.58	3.301 (3)	131
C21—H21⋯O6^v^	0.93	2.52	3.302 (4)	143

Symmetry codes: (i) 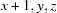; (ii) 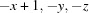; (iii) 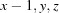; (iv) 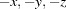; (v) 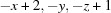.

**Fig. 1.**
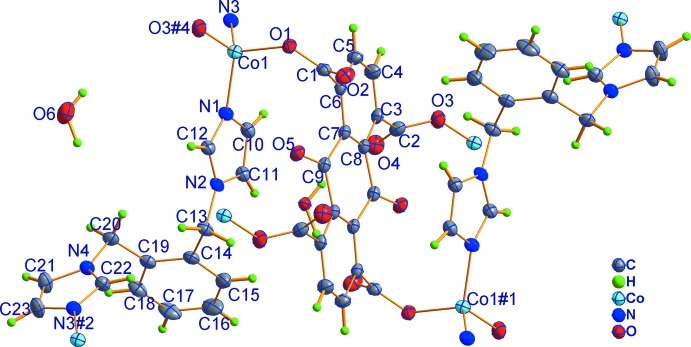
State of an asymmetric unit of compound (I), showing the atom-numbering scheme and displacement ellipsoids at the 30% probability level(Symmetry code: #1 - x,-y,-z; #2 x,y - 1,z; #3 x,y + 1,z; #4 x + 1,y,z; #5 x - 1,y,z).

**Fig. 2.**
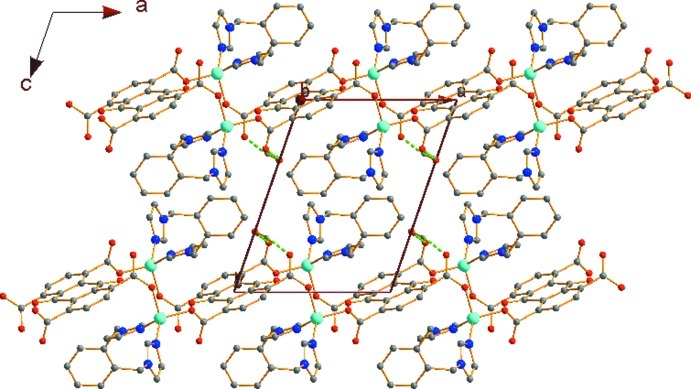
: A view of stacking structure of the title compound (H atom omited for clear except solvent water).

## supplementary crystallographic information

### Comment

Porous solid materials, such as MOFs (metal-organic frameworks) have been widely studied for their potential applications in gas absorption, separation, catalysis and magnetic materials. explorations of advanced porous materials for these applications are an intense subject of scientific research. (Li *et al.*, 1999; Li *et al.*, 2012; Cheng *et al.*, 2010; Hong *et al.*, 2009; Miller *et al.*, 2011; Liu *et al.*, 2010.) Herein we report the crystal structure of the title compound.

The molecular structure of (I) is illustrated in Fig. 1., a summary of the observed hydrogen bonds and the corresponding angles are given in Table 1.

The center of the anthraquinone moiety is a crystallographic center of inversion. Each asymmetric unit therefore contains one cobalt atom and one *o*-bix ligand as well as one half AQTC^4-^ ligand and an additional solvent water molecule. Cobalt(II) ions are tetrahedrally surrounded by two O atoms from two AQTC^4-^ and two N atoms from two *o*-bix ligands forming a 2D-coordination polymer.

### Experimental

A mixture of H_4_AQTC (0.025 mmol, 9.8 mg) and *o*-bix (0.025 mmol, 6.0 mg) were added to distilled water (4 ml) and ultra-sounded for 10 min. The pH value of the mixture was then adjusted to 8.0 with NaOH (0.5 mol.*L*^-1^). Then CoCl_2_ × 6 H_2_O (0.05 mmol, 12 mg) was added. The reactants were placed in a Teflon-lined stainless steel vessel, heated for 3 days, and then cooled to ambient temperature over 12 h. Red block shaped crystals of (I) were obtained in 30% yield.

### Refinement

All non-hydrogen atoms were refined anisotropically. H atoms of the H_2_O were located from difference Fourier maps and refined isotropically with a distance restraint of O-H = 0.83-0.99Å. Carbon bound H atoms were placed in calculated positionswith C-H = 0.93 Å for aromatic and 0.97 Å for methylene hydrogen atoms and refined as riding atoms, with U_iso_(H) = 1.2U_eq_(C).

### Crystal data

**Table:**

[Co_2_(C_18_H_4_O_10_)(C_14_H_14_N_4_)_2_]·H_2_O	*Z* = 1
*M**_r_* = 1010.68	*F*(000) = 518
Triclinic, *P*1	*D*_x_ = 1.539 Mg m^−^^3^
Hall symbol: -P 1	Mo *K*α radiation, λ = 0.71073 Å
*a* = 9.561 (4) Å	Cell parameters from 5133 reflections
*b* = 10.594 (5) Å	θ = 2.2–27.6°
*c* = 12.436 (5) Å	µ = 0.83 mm^−^^1^
α = 107.095 (7)°	*T* = 296 K
β = 102.454 (6)°	Block, red
γ = 106.551 (6)°	0.43 × 0.36 × 0.28 mm
*V* = 1090.7 (8) Å^3^	

### Data collection

**Table:**

Bruker SMART CCD area-detector diffractometer	3787 independent reflections
Radiation source: sealed tube	3411 reflections with *I* > 2σ(*I*)
Graphite monochromator	*R*_int_ = 0.045
phi and ω scans	θ_max_ = 25.0°, θ_min_ = 1.8°
Absorption correction: multi-scan (*SADABS*; Bruker, 2000)	*h* = −11→11
*T*_min_ = 0.717, *T*_max_ = 0.801	*k* = −12→12
10020 measured reflections	*l* = −14→14

### Refinement

**Table:**

Refinement on *F*^2^	Primary atom site location: structure-invariant direct methods
Least-squares matrix: full	Secondary atom site location: difference Fourier map
*R*[*F*^2^ > 2σ(*F*^2^)] = 0.032	Hydrogen site location: inferred from neighbouring sites
*wR*(*F*^2^) = 0.091	H atoms treated by a mixture of independent and constrained refinement
*S* = 1.04	*w* = 1/[σ^2^(*F*_o_^2^) + (0.0366*P*)^2^ + 0.4627*P*] where *P* = (*F*_o_^2^ + 2*F*_c_^2^)/3
3787 reflections	(Δ/σ)_max_ = 0.001
315 parameters	Δρ_max_ = 0.39 e Å^−^^3^
0 restraints	Δρ_min_ = −0.32 e Å^−^^3^

### Special details

**Table:**

Experimental. Anal. Calcd. for C_23_H_18_Co_1_N_4_O_6_: C, 54.66; H, 3.59; N, 11.09%. Found: C, 54.34; H, 3.37; N, 10.86%. FT—IR data (KBr pellets, cm^-1^): 3359(*m*), 3140(w), 3111(*m*), 15109(*m*), 1455(w), 1078(*m*), 754(*m*), 732(w), 685(*m*).
Geometry. All e.s.d.'s (except the e.s.d. in the dihedral angle between two l.s. planes) are estimated using the full covariance matrix. The cell e.s.d.'s are taken into account individually in the estimation of e.s.d.'s in distances, angles and torsion angles; correlations between e.s.d.'s in cell parameters are only used when they are defined by crystal symmetry. An approximate (isotropic) treatment of cell e.s.d.'s is used for estimating e.s.d.'s involving l.s. planes.
Refinement. Refinement of *F*^2^ against ALL reflections. The weighted *R*-factor *wR* and goodness of fit *S* are based on *F*^2^, conventional *R*-factors *R* are based on *F*, with *F* set to zero for negative *F*^2^. The threshold expression of *F*^2^ > σ(*F*^2^) is used only for calculating *R*-factors(gt) *etc*. and is not relevant to the choice of reflections for refinement. *R*-factors based on *F*^2^ are statistically about twice as large as those based on *F*, and *R*- factors based on ALL data will be even larger.

### Fractional atomic coordinates and isotropic or equivalent isotropic displacement parameters (Å^2^)

**Table:**

	*x*	*y*	*z*	*U*_iso_*/*U*_eq_	
C1	0.3229 (3)	0.2312 (2)	−0.08418 (19)	0.0307 (5)	
C2	−0.1263 (3)	0.3036 (2)	0.1570 (2)	0.0329 (5)	
C3	−0.0113 (3)	0.2767 (2)	0.09487 (18)	0.0301 (5)	
C4	0.0924 (3)	0.3922 (2)	0.0854 (2)	0.0377 (5)	
H4	0.0902	0.4823	0.1182	0.045*	
C5	0.1986 (3)	0.3753 (2)	0.0281 (2)	0.0362 (5)	
H5	0.2643	0.4533	0.0203	0.043*	
C6	0.2080 (3)	0.2425 (2)	−0.01795 (18)	0.0286 (5)	
C7	0.1067 (2)	0.1253 (2)	−0.00744 (17)	0.0258 (4)	
C8	−0.0065 (2)	0.1415 (2)	0.04555 (17)	0.0262 (4)	
C9	0.1271 (2)	−0.0137 (2)	−0.04162 (17)	0.0263 (4)	
C10	0.3621 (3)	0.1884 (2)	0.23544 (18)	0.0307 (5)	
H10	0.3323	0.2661	0.2552	0.037*	
C11	0.2936 (3)	0.0631 (2)	0.24410 (18)	0.0295 (5)	
H11	0.2085	0.0380	0.2692	0.035*	
C12	0.4870 (3)	0.0555 (2)	0.17763 (18)	0.0302 (5)	
H12	0.5576	0.0225	0.1495	0.036*	
C13	0.3365 (3)	−0.1710 (2)	0.19313 (19)	0.0345 (5)	
H13A	0.4237	−0.1977	0.1848	0.041*	
H13B	0.2499	−0.2302	0.1207	0.041*	
C14	0.2956 (3)	−0.1986 (2)	0.29720 (19)	0.0319 (5)	
C15	0.1425 (3)	−0.2315 (3)	0.2937 (3)	0.0468 (6)	
H15	0.0691	−0.2373	0.2279	0.056*	
C16	0.0982 (4)	−0.2559 (3)	0.3875 (3)	0.0635 (9)	
H16	−0.0041	−0.2765	0.3847	0.076*	
C17	0.2054 (4)	−0.2496 (3)	0.4845 (3)	0.0601 (9)	
H17	0.1758	−0.2662	0.5471	0.072*	
C18	0.3566 (3)	−0.2188 (3)	0.4884 (2)	0.0442 (6)	
H18	0.4283	−0.2161	0.5536	0.053*	
C19	0.4047 (3)	−0.1913 (2)	0.39615 (19)	0.0305 (5)	
C20	0.5728 (3)	−0.1577 (2)	0.4074 (2)	0.0344 (5)	
H20A	0.5976	−0.1098	0.3545	0.041*	
H20B	0.6361	−0.0934	0.4882	0.041*	
C21	0.6999 (3)	−0.3245 (3)	0.4543 (2)	0.0467 (6)	
H21	0.7494	−0.2738	0.5356	0.056*	
C22	0.5629 (3)	0.6109 (2)	0.26958 (19)	0.0337 (5)	
H22	0.5005	0.6117	0.2013	0.040*	
C23	0.7029 (3)	0.5502 (3)	0.3882 (2)	0.0494 (7)	
H23	0.7557	0.4994	0.4172	0.059*	
Co1	0.58835 (3)	0.33412 (3)	0.13769 (2)	0.02845 (12)	
N1	0.4836 (2)	0.18355 (19)	0.19274 (15)	0.0304 (4)	
N2	0.3755 (2)	−0.02007 (18)	0.20818 (15)	0.0281 (4)	
N3	0.6159 (2)	0.50944 (18)	0.27147 (16)	0.0315 (4)	
N4	0.6099 (2)	−0.28740 (18)	0.37783 (15)	0.0299 (4)	
O1	0.46596 (18)	0.30258 (17)	−0.02288 (13)	0.0351 (4)	
O2	0.2749 (2)	0.16438 (19)	−0.19247 (14)	0.0458 (4)	
O3	−0.22850 (19)	0.33483 (18)	0.09643 (15)	0.0395 (4)	
O4	−0.1135 (2)	0.29987 (19)	0.25620 (15)	0.0463 (4)	
O5	0.24715 (17)	−0.02029 (16)	−0.05752 (13)	0.0331 (4)	
O6	0.9984 (3)	0.0915 (3)	0.3137 (2)	0.0614 (6)	
H25	0.912 (6)	−0.001 (6)	0.285 (5)	0.15 (2)*	
H24	0.982 (5)	0.161 (5)	0.304 (4)	0.108 (17)*	

### Atomic displacement parameters (Å^2^)

**Table:**

	*U*^11^	*U*^22^	*U*^33^	*U*^12^	*U*^13^	*U*^23^
C1	0.0325 (14)	0.0336 (11)	0.0336 (11)	0.0138 (10)	0.0166 (10)	0.0177 (9)
C2	0.0306 (13)	0.0294 (10)	0.0355 (12)	0.0101 (10)	0.0150 (10)	0.0060 (9)
C3	0.0253 (12)	0.0362 (11)	0.0287 (10)	0.0134 (9)	0.0093 (9)	0.0100 (9)
C4	0.0377 (15)	0.0327 (11)	0.0452 (13)	0.0163 (10)	0.0185 (11)	0.0115 (10)
C5	0.0343 (14)	0.0347 (11)	0.0417 (12)	0.0115 (10)	0.0169 (11)	0.0156 (10)
C6	0.0246 (12)	0.0375 (11)	0.0260 (10)	0.0129 (9)	0.0095 (9)	0.0131 (9)
C7	0.0227 (12)	0.0346 (10)	0.0226 (9)	0.0123 (9)	0.0096 (8)	0.0109 (8)
C8	0.0236 (12)	0.0342 (11)	0.0235 (10)	0.0129 (9)	0.0097 (8)	0.0111 (8)
C9	0.0239 (12)	0.0368 (11)	0.0198 (9)	0.0123 (9)	0.0098 (8)	0.0099 (8)
C10	0.0369 (13)	0.0357 (11)	0.0288 (10)	0.0204 (10)	0.0158 (10)	0.0147 (9)
C11	0.0325 (13)	0.0379 (11)	0.0277 (10)	0.0183 (10)	0.0164 (9)	0.0157 (9)
C12	0.0330 (13)	0.0370 (11)	0.0299 (11)	0.0175 (10)	0.0166 (10)	0.0164 (9)
C13	0.0487 (15)	0.0312 (11)	0.0289 (11)	0.0174 (10)	0.0166 (10)	0.0135 (9)
C14	0.0412 (14)	0.0271 (10)	0.0342 (11)	0.0156 (10)	0.0180 (10)	0.0144 (9)
C15	0.0380 (16)	0.0479 (14)	0.0630 (16)	0.0172 (12)	0.0190 (13)	0.0297 (13)
C16	0.052 (2)	0.074 (2)	0.100 (2)	0.0316 (16)	0.0545 (19)	0.0523 (19)
C17	0.082 (2)	0.0705 (19)	0.0719 (19)	0.0419 (18)	0.0586 (19)	0.0506 (16)
C18	0.0666 (19)	0.0486 (14)	0.0380 (13)	0.0318 (13)	0.0300 (13)	0.0252 (11)
C19	0.0419 (14)	0.0270 (10)	0.0304 (11)	0.0170 (10)	0.0189 (10)	0.0125 (8)
C20	0.0415 (15)	0.0262 (10)	0.0347 (11)	0.0146 (10)	0.0132 (10)	0.0081 (9)
C21	0.0584 (18)	0.0495 (14)	0.0289 (12)	0.0294 (14)	0.0056 (11)	0.0077 (10)
C22	0.0423 (15)	0.0307 (11)	0.0283 (11)	0.0150 (10)	0.0114 (10)	0.0105 (9)
C23	0.0604 (19)	0.0484 (15)	0.0432 (14)	0.0346 (14)	0.0079 (13)	0.0150 (12)
Co1	0.0323 (2)	0.02908 (17)	0.03102 (17)	0.01470 (14)	0.01786 (14)	0.01245 (13)
N1	0.0347 (11)	0.0334 (9)	0.0308 (9)	0.0162 (8)	0.0165 (8)	0.0152 (8)
N2	0.0346 (11)	0.0326 (9)	0.0261 (8)	0.0169 (8)	0.0153 (8)	0.0150 (7)
N3	0.0356 (11)	0.0304 (9)	0.0335 (9)	0.0159 (8)	0.0167 (8)	0.0119 (8)
N4	0.0353 (11)	0.0284 (9)	0.0274 (9)	0.0147 (8)	0.0118 (8)	0.0088 (7)
O1	0.0277 (10)	0.0480 (9)	0.0394 (8)	0.0151 (8)	0.0176 (7)	0.0243 (7)
O2	0.0449 (11)	0.0543 (10)	0.0340 (9)	0.0097 (9)	0.0214 (8)	0.0136 (8)
O3	0.0338 (10)	0.0504 (10)	0.0462 (9)	0.0245 (8)	0.0216 (8)	0.0201 (8)
O4	0.0502 (12)	0.0581 (11)	0.0356 (9)	0.0248 (9)	0.0224 (8)	0.0143 (8)
O5	0.0267 (9)	0.0384 (8)	0.0385 (8)	0.0149 (7)	0.0185 (7)	0.0122 (7)
O6	0.0451 (14)	0.0713 (15)	0.0586 (13)	0.0237 (12)	0.0117 (10)	0.0142 (11)

### Geometric parameters (Å, º)

**Table:**

C1—O2	1.230 (3)	C14—C19	1.398 (3)
C1—O1	1.283 (3)	C15—C16	1.392 (4)
C1—C6	1.519 (3)	C15—H15	0.9300
C2—O4	1.226 (3)	C16—C17	1.374 (5)
C2—O3	1.283 (3)	C16—H16	0.9300
C2—C3	1.517 (3)	C17—C18	1.374 (4)
C3—C4	1.392 (3)	C17—H17	0.9300
C3—C8	1.401 (3)	C18—C19	1.400 (3)
C4—C5	1.382 (3)	C18—H18	0.9300
C4—H4	0.9300	C19—C20	1.509 (3)
C5—C6	1.391 (3)	C20—N4	1.478 (3)
C5—H5	0.9300	C20—H20A	0.9700
C6—C7	1.401 (3)	C20—H20B	0.9700
C7—C8	1.408 (3)	C21—C23^ii^	1.352 (3)
C7—C9	1.489 (3)	C21—N4	1.364 (3)
C8—C9^i^	1.488 (3)	C21—H21	0.9300
C9—O5	1.223 (3)	C22—N3	1.316 (3)
C9—C8^i^	1.488 (3)	C22—N4^iii^	1.336 (3)
C10—C11	1.349 (3)	C22—H22	0.9300
C10—N1	1.384 (3)	C23—C21^iii^	1.352 (3)
C10—H10	0.9300	C23—N3	1.376 (3)
C11—N2	1.380 (3)	C23—H23	0.9300
C11—H11	0.9300	Co1—O3^iv^	1.9267 (17)
C12—N1	1.325 (3)	Co1—O1	1.9610 (17)
C12—N2	1.337 (3)	Co1—N3	2.0008 (18)
C12—H12	0.9300	Co1—N1	2.0110 (18)
C13—N2	1.479 (3)	N4—C22^ii^	1.336 (3)
C13—C14	1.513 (3)	O3—Co1^v^	1.9267 (17)
C13—H13A	0.9700	O6—H25	0.99 (6)
C13—H13B	0.9700	O6—H24	0.83 (5)
C14—C15	1.392 (4)		
			
O2—C1—O1	124.1 (2)	C17—C16—C15	120.1 (3)
O2—C1—C6	119.3 (2)	C17—C16—H16	119.9
O1—C1—C6	116.41 (18)	C15—C16—H16	119.9
O4—C2—O3	126.4 (2)	C16—C17—C18	119.7 (2)
O4—C2—C3	121.2 (2)	C16—C17—H17	120.2
O3—C2—C3	112.36 (19)	C18—C17—H17	120.2
C4—C3—C8	118.88 (19)	C17—C18—C19	121.3 (3)
C4—C3—C2	118.03 (19)	C17—C18—H18	119.3
C8—C3—C2	123.08 (19)	C19—C18—H18	119.3
C5—C4—C3	121.2 (2)	C14—C19—C18	119.0 (2)
C5—C4—H4	119.4	C14—C19—C20	122.88 (19)
C3—C4—H4	119.4	C18—C19—C20	118.1 (2)
C4—C5—C6	120.5 (2)	N4—C20—C19	111.96 (18)
C4—C5—H5	119.8	N4—C20—H20A	109.2
C6—C5—H5	119.8	C19—C20—H20A	109.2
C5—C6—C7	119.27 (19)	N4—C20—H20B	109.2
C5—C6—C1	117.40 (19)	C19—C20—H20B	109.2
C7—C6—C1	123.23 (19)	H20A—C20—H20B	107.9
C6—C7—C8	120.01 (19)	C23^ii^—C21—N4	106.3 (2)
C6—C7—C9	120.37 (18)	C23^ii^—C21—H21	126.9
C8—C7—C9	119.38 (18)	N4—C21—H21	126.9
C3—C8—C7	119.98 (19)	N3—C22—N4^iii^	111.6 (2)
C3—C8—C9^i^	120.16 (18)	N3—C22—H22	124.2
C7—C8—C9^i^	119.73 (18)	N4^iii^—C22—H22	124.2
O5—C9—C8^i^	120.24 (19)	C21^iii^—C23—N3	109.5 (2)
O5—C9—C7	120.15 (19)	C21^iii^—C23—H23	125.2
C8^i^—C9—C7	119.39 (18)	N3—C23—H23	125.2
C11—C10—N1	109.68 (19)	O3^iv^—Co1—O1	94.24 (7)
C11—C10—H10	125.2	O3^iv^—Co1—N3	115.00 (8)
N1—C10—H10	125.2	O1—Co1—N3	117.63 (8)
C10—C11—N2	105.96 (19)	O3^iv^—Co1—N1	119.89 (8)
C10—C11—H11	127.0	O1—Co1—N1	111.05 (7)
N2—C11—H11	127.0	N3—Co1—N1	100.17 (8)
N1—C12—N2	111.04 (19)	C12—N1—C10	105.58 (17)
N1—C12—H12	124.5	C12—N1—Co1	131.62 (14)
N2—C12—H12	124.5	C10—N1—Co1	121.56 (14)
N2—C13—C14	112.12 (16)	C12—N2—C11	107.74 (18)
N2—C13—H13A	109.2	C12—N2—C13	125.85 (18)
C14—C13—H13A	109.2	C11—N2—C13	126.09 (18)
N2—C13—H13B	109.2	C22—N3—C23	105.27 (18)
C14—C13—H13B	109.2	C22—N3—Co1	129.70 (16)
H13A—C13—H13B	107.9	C23—N3—Co1	125.00 (16)
C15—C14—C19	119.1 (2)	C22^ii^—N4—C21	107.31 (18)
C15—C14—C13	118.1 (2)	C22^ii^—N4—C20	125.81 (19)
C19—C14—C13	122.8 (2)	C21—N4—C20	126.87 (18)
C14—C15—C16	120.7 (3)	C1—O1—Co1	132.29 (13)
C14—C15—H15	119.6	C2—O3—Co1^v^	120.57 (14)
C16—C15—H15	119.6	H25—O6—H24	120 (4)
			
O4—C2—C3—C4	−107.8 (3)	C17—C18—C19—C14	−1.4 (4)
O3—C2—C3—C4	69.5 (3)	C17—C18—C19—C20	179.8 (2)
O4—C2—C3—C8	73.1 (3)	C14—C19—C20—N4	−100.3 (2)
O3—C2—C3—C8	−109.6 (2)	C18—C19—C20—N4	78.5 (2)
C8—C3—C4—C5	0.3 (4)	N2—C12—N1—C10	0.0 (2)
C2—C3—C4—C5	−178.9 (2)	N2—C12—N1—Co1	−167.10 (15)
C3—C4—C5—C6	−2.3 (4)	C11—C10—N1—C12	−0.7 (2)
C4—C5—C6—C7	1.1 (3)	C11—C10—N1—Co1	168.03 (15)
C4—C5—C6—C1	177.5 (2)	O3^iv^—Co1—N1—C12	−21.8 (2)
O2—C1—C6—C5	−110.7 (2)	O1—Co1—N1—C12	86.4 (2)
O1—C1—C6—C5	64.2 (3)	N3—Co1—N1—C12	−148.5 (2)
O2—C1—C6—C7	65.6 (3)	O3^iv^—Co1—N1—C10	172.85 (15)
O1—C1—C6—C7	−119.5 (2)	O1—Co1—N1—C10	−78.94 (17)
C5—C6—C7—C8	2.2 (3)	N3—Co1—N1—C10	46.09 (18)
C1—C6—C7—C8	−174.02 (19)	N1—C12—N2—C11	0.7 (2)
C5—C6—C7—C9	−172.1 (2)	N1—C12—N2—C13	174.39 (19)
C1—C6—C7—C9	11.7 (3)	C10—C11—N2—C12	−1.0 (2)
C4—C3—C8—C7	3.0 (3)	C10—C11—N2—C13	−174.75 (19)
C2—C3—C8—C7	−177.9 (2)	C14—C13—N2—C12	141.3 (2)
C4—C3—C8—C9^i^	−172.8 (2)	C14—C13—N2—C11	−46.2 (3)
C2—C3—C8—C9^i^	6.2 (3)	N4^iii^—C22—N3—C23	−0.5 (3)
C6—C7—C8—C3	−4.3 (3)	N4^iii^—C22—N3—Co1	−178.48 (14)
C9—C7—C8—C3	170.09 (19)	C21^iii^—C23—N3—C22	0.2 (3)
C6—C7—C8—C9^i^	171.61 (19)	C21^iii^—C23—N3—Co1	178.31 (18)
C9—C7—C8—C9^i^	−14.0 (3)	O3^iv^—Co1—N3—C22	109.9 (2)
C6—C7—C9—O5	13.8 (3)	O1—Co1—N3—C22	0.3 (2)
C8—C7—C9—O5	−160.54 (19)	N1—Co1—N3—C22	−120.1 (2)
C6—C7—C9—C8^i^	−171.68 (18)	O3^iv^—Co1—N3—C23	−67.7 (2)
C8—C7—C9—C8^i^	14.0 (3)	O1—Co1—N3—C23	−177.34 (19)
N1—C10—C11—N2	1.1 (2)	N1—Co1—N3—C23	62.3 (2)
N2—C13—C14—C15	88.0 (3)	C23^ii^—C21—N4—C22^ii^	−0.4 (3)
N2—C13—C14—C19	−91.5 (3)	C23^ii^—C21—N4—C20	−179.3 (2)
C19—C14—C15—C16	0.5 (4)	C19—C20—N4—C22^ii^	69.5 (3)
C13—C14—C15—C16	−179.1 (2)	C19—C20—N4—C21	−111.9 (3)
C14—C15—C16—C17	−0.9 (4)	O2—C1—O1—Co1	−143.51 (19)
C15—C16—C17—C18	0.2 (5)	C6—C1—O1—Co1	41.9 (3)
C16—C17—C18—C19	1.0 (4)	O3^iv^—Co1—O1—C1	144.39 (19)
C15—C14—C19—C18	0.6 (3)	N3—Co1—O1—C1	−94.5 (2)
C13—C14—C19—C18	−179.8 (2)	N1—Co1—O1—C1	20.1 (2)
C15—C14—C19—C20	179.4 (2)	O4—C2—O3—Co1^v^	−16.1 (3)
C13—C14—C19—C20	−1.1 (3)	C3—C2—O3—Co1^v^	166.74 (14)

Symmetry codes: (i) −*x*, −*y*, −*z*; (ii) *x*, *y*−1, *z*; (iii) *x*, *y*+1, *z*; (iv) *x*+1, *y*, *z*; (v) *x*−1, *y*, *z*.

### Hydrogen-bond geometry (Å, º)

**Table:**

*D*—H···*A*	*D*—H	H···*A*	*D*···*A*	*D*—H···*A*
O6—H24···O4^iv^	0.83 (5)	2.10 (5)	2.911 (4)	166 (5)
O6—H25···O2^vi^	0.99 (6)	1.92 (6)	2.883 (4)	164 (5)
C11—H11···O6^v^	0.93	2.37	3.192 (4)	148
C12—H12···O5^vi^	0.93	2.39	3.268 (4)	157
C13—H13*A*···O1^vi^	0.97	2.58	3.389 (4)	141
C13—H13*B*···O3^i^	0.97	2.54	3.278 (3)	133
C20—H20*A*···O2^vi^	0.97	2.58	3.301 (3)	131
C21—H21···O6^vii^	0.93	2.52	3.302 (4)	143

Symmetry codes: (i) −*x*, −*y*, −*z*; (iv) *x*+1, *y*, *z*; (v) *x*−1, *y*, *z*; (vi) −*x*+1, −*y*, −*z*; (vii) −*x*+2, −*y*, −*z*+1.
